# Hospitalizations for Substance Abuse Disorders Before and After Hurricane Katrina: Spatial Clustering and Area-Level Predictors, New Orleans, 2004 and 2008

**DOI:** 10.5888/pcd13.160107

**Published:** 2016-10-13

**Authors:** Imelda K. Moise, Marilyn O. Ruiz

**Affiliations:** Author Affiliation: Marilyn O. Ruiz, Department of Pathobiology, University of Illinois at Urbana-Champaign, Urbana, Illinois.

## Abstract

**Introduction:**

Identifying at-risk groups is a challenge in post-disaster psychosocial response. Geospatial techniques can support the design and deployment of targeted and tailored interventions. This study compared spatial patterns in the distribution of hospitalizations for substance abuse disorders and associated area-level predictors before and after Hurricane Katrina in New Orleans, Louisiana.

**Methods:**

We used hospital data from the Louisiana Department of Health and Hospitals for 2004 (pre-Katrina) and 2008 (post-Katrina). Data were assessed by using descriptive statistics, multivariable Poisson regression, and geospatial analysis. We assessed hospitalizations by US Census block group in relation to the presence of blighted properties (ie, buildings declared an imminent health threat, in danger of collapse, or a public nuisance), race of residents (white or nonwhite), presence of nondisplaced residents (measured by the number of households receiving mail in 2008), and depth of water levels.

**Results:**

The hospitalization rate for substance abuse disorders was 7.13 per 1,000 population for 2004 and 9.65 per 1,000 for 2008. The concentration of hospitalizations shifted geographically from block groups exposed to floods (levee breaches) in 2004 to the center of the city in 2008. Post Katrina, predictors for hospitalizations were presence of blighted properties, nonwhite populations, and presence of nondisplaced residents. Distance from flooded areas (high water depth) and levee breaches was negatively associated with hospitalizations. Men were more likely than women to be hospitalized during both periods (78%, 2004; 63%, 2008), and the percentage of the hospitalized white population increased from 2004 (28.8%) to 2008 (44.9%).

**Conclusion:**

Geographic patterns of hospitalizations for substance abuse disorders shifted in post-Katrina New Orleans from flood-exposed areas to less exposed areas in the center of the city; however, poverty was a main predictor for hospitalizations during both periods. Approaches used in this study are generalizable to other disaster areas and to other psychological vulnerabilities (eg, anxiety).

## Introduction

Consensus is emerging among disaster researchers that psychological disorders and substance abuse increases in the aftermath of both man-made and natural disasters (1–8). Exposure to a disaster can entail physical threats to life and post-disaster behavior and re-adjustment problems (eg, dealing with loss of home, friends, or family). These events can increase the risk of substance abuse, such as extensive drinking or drug use, as a coping mechanism (9–13). The risk for substance abuse in the aftermath of disasters is likely to vary among residential areas. Also, the extent to which area-level risk factors for substance abuse is associated with post-disaster substance abuse is likely to vary across residential contexts. This presents challenges to mental health professionals who have to identify the affected and at-risk groups in exposed communities to provide tailored interventions and to meet their needs in a timely manner.

Spatial analysis and geographical information systems (GIS) software can be used in the post-disaster context. These techniques facilitate identification of at-risk areas, at-risk populations, service gaps, and associated residential risk factors (14,15). The information generated from application of these techniques can help local governments to design and implement targeted interventions. Targeted interventions enable a more efficient allocation of treatment and prevention resources and better decisions about treatment of substance abuse disorders through improved access to mental health services.

Despite the availability of research tools and techniques, few studies have investigated post-disaster psychological outcomes geographically, although recent evidence suggests that increased proximity to a disaster event is associated with increased negative mental health outcomes (eg, posttraumatic stress, depression, anxiety-related disorders, prolonged grief) (16–19). Few studies to date compared pre- and post-disaster substance abuse disorders to distinguish changes in these disorders among individuals or communities with varying degrees of disaster exposures (20). Even less is known about the increased likelihood of hospitalization for substance abuse disorders. Additionally, research to date has focused on the use of local surveys rather than fine-grained, geographically refined hospital-based data.

Our study’s objective was to use spatial analysis techniques, GIS, and fine-grained hospitalization data to identify geographic patterns in substance abuse disorders in a post-disaster setting. The study aimed 1) to describe geographical variation in hospitalizations for substance abuse disorders in New Orleans before and after Hurricane Katrina, and 2) to determine whether these differences were independent of known risk factors for such hospitalizations. We hypothesized that hospitalizations for substance abuse disorders would cluster predominantly in block groups that were most heavily exposed to the physical effects of the hurricane.

## Methods

### Data sources

**Hospitalization data**. We examined hospital data for 2004 and 2008 on substance abuse disorders from the Louisiana Department of Health and Hospital’s Bureau of Policy Research and Health Systems Analysis. These 2 years were selected to reflect conditions before and after the extensive wind and flood damage caused by Hurricane Katrina in August 2005. This data set collates hospitalization records from private and public hospitals in the state, excluding Veteran Affairs hospitals. Diagnosis categories for substance abuse disorders were based on the Single Layer Clinical Classification System (SLCCS) (https://www.hcup-us.ahrq.gov), which groups all ICD-9 (*International Classification of Diseases, 9th Revision*) diagnosis codes into 2 clinically meaningful, mutually exclusive diagnosis categories (SLCCS code 66, alcohol-related mental disorders; SLCCS code 67, substance-related mental disorders) (Appendix). The primary sources for the requested data were the UB-04 claim forms of the American Health Information Management Association Clinical Terminology and Classification Practice Council 2007 (http://library.ahima.org/doc?oid=99893#.V7-kDSgrKhc).

We used data from the 2000 US Census to measure the pre-Katrina population of New Orleans. In July 2005, before Katrina struck, Orleans Parish (Orleans Parish and the city of New Orleans are coterminous) had a population of 437,186. We then used 2008 population data from GRC and Associates Inc (http://www.grcassoc.com/) to estimate the city’s 2008 post-Katrina population of 235,651 residents. Estimates for 2008 were based on indicators of residential occupancy (eg, utility accounts, voter registration) calibrated against base population data from the 2000 census. In 2008, these estimates were the most reliable post-Katrina estimates of the New Orleans population. Socioeconomic data at the block group level were obtained from the 2005–2009 US Census American Community Survey (ACS). These data included median year a house was built, median household income, population density, vacant properties, race (white or nonwhite), population using public assistance, population living below the federal poverty level, and change in the number of households from 2005 through 2009 (year 2000 households compared with households in ACS 2005–2009). Neighborhood recovery was assessed by using data on number of nondisplaced residents measured as households receiving mail in 2008, a proxy for the number of residences that were inhabited in 2008. These data were obtained from the Greater New Orleans Community Data Center, which originally purchased the 2009 data set from Valassis Communications, Inc (www.valassis.com/).

We assessed neighborhood predictors as the number of blighted properties (ie, buildings declared an imminent health threat, in danger of collapse, or a public nuisance) and neighborhood recovery (completed property demolitions) by using lists of address-level data for years 2006–2009. This information was obtained from the City of New Orleans Department of Code Enforcement (http://www.nola.gov/). To capture data on the physical impact of Hurricane Katrina, we summarized mean water depths measured during the days immediately after the storm for each block group by using the Spatial Analyst tools feature in ArcGIS version 10.3.1 (Esri Inc). Water depths were based on original data provided by the National Oceanic and Atmospheric Administration.

### Analysis

To examine the presence of clusters of hospitalizations for substance abuse disorders, we applied SaTScan version 9.4 (SaTScan) by using a retrospective, spatial analysis and the discrete Poisson probability model (21) with covariates (age group and sex). To observe change over time, we performed cluster analysis separately on the 2 years (2004 and 2008) and used the same block groups for both periods. The population counts included in this analysis were from the 2000 US Census and the 2008 population estimate of GRC and Associates Inc. We assumed that the number of substance abuse disorders in each block group was Poisson-distributed. The level of significance used for this study was *P* = .05. We tested the null hypothesis that the relative risk of hospitalizations for substance abuse disorders was the same inside as outside the scanning window. To avoid selection bias, we allowed the statistics to scan areas (impose a circular window on the map) with varying sizes. SaTScan software is designed to implement this test by allowing a window to move over the study area and center on the centroid of each block group. The most likely cluster (the cluster least likely to be due to chance) is assigned to a window with the maximum likelihood. Cluster analysis was performed with the default maximum spatial cluster size of less than 50% of the at-risk population. We determined both the most likely clusters and secondary clusters that cause a rejection of the null hypothesis and do not overlap the most likely cluster. Results were mapped by block groups using ArcGIS version 10.3.1.

Descriptive statistics were used to characterize people hospitalized with substance abuse disorders. To examine the extent to which neighborhood predictors were associated over time with these hospitalizations, we used Poisson regression (generalized linear models) with Poisson distribution and a log link function with random effects for block group and an offset for the population. The Poisson model was appropriate because the response variable is a count (number of hospitalizations for substance abuse disorders per block group), and the counts were small. The Poisson regression model was developed by adding potential predictors with their time interactions one by one and retaining those that had significant effects. To account for correlations between hospitalizations resulting from unmodeled variation between block groups, a normally distributed random effect was added for each block group; to make decisions about which predictors to maintain, Wald tests, *F* tests, and information criteria (Akaike information criterion and Bayesian information criteria methods) were used. Of the 13 variables used in the neighborhood regression analysis, 7 were retained (number of housing units, percentage of population living below the federal poverty level, percentage of population that was white, blighted properties, number of buildings demolished, water depth, and number of households receiving mail).

## Results

From 2004 through 2008, 1,401 people aged 1 to 97 years were hospitalized with a substance abuse disorder in hospitals in the Greater New Orleans area (New Orleans–Metairie-Kenner Metropolitan Statistical Area). Pre-Katrina, the annual hospitalization rate was 7.13 per 1,000, and post-Katrina the rate was 9.65 per 1,000. The variation in age of those hospitalized was greater in the post-Katrina period than in the pre-Katrina period (standard deviation [SD], 12 vs SD, 13). Post-Katrina hospitalizations were spread across age groups more evenly, but pre-Katrina, most hospitalizations were clustered in the 20-to-49 age group. Men were more likely to be hospitalized than women in both periods but somewhat less so post-Katrina (78% of all hospitalizations for psychological disorders [including anxiety disorders and psychosomatic disorders] in 2004 and 63% in 2008). The proportion of white patients increased from 28.8% in 2004 to 44.9% in 2008, (*P* < .001, χ^2^ test). Table 1 presents demographic characteristics of the 13 New Orleans planning districts with patients hospitalized for substance abuse disorders in 2004 and 2008.

**Table 1:** Demographic Characteristics of New Orleans Planning Districts (n = 13) With Patients Hospitalized for Substance Abuse Disorders, 2004 and 2008

Planning District	Number of Substance Abuse Disorders		Population		Average Age		Average Percentage White		Average Percentage Living in Poverty^a^		Substance Abuse Disorders, Rate Per 1,000 Population	
	2004	2008	2004	2008	2004	2008	2004	2008	2004	2008	2004	2008
Algiers	71	61	50,022	48,222	38	36	30.3	21	34.7	31.1	0.6	0.6
Bywater	81	81	33,738	22,375	36	40	18.6	12.9	34.1	33.7	1	1.8
Garden District	90	105	30,800	32,636	42	43	31.6	20.6	43.4	44.6	1.3	1.6
French Quarter/ Central Business District	49	34	15,873	8,269	40	40	69	74.3	72.5	71.4	1.3	2.1
Gentilly	47	34	23,620	8,896	39	38	14.2	15.2	35.5	36.2	1	1.9
Lakeview	36	16	16,893	5,281	43	37	67.1	80.5	46.6	43.2	1.1	1.5
Lower 9th Ward	34	14	13,250	1,209	38	42	4	1.5	36.7	32.7	1.2	5.8
Mid-City	150	127	100,140	37,006	38	41	12.6	14	34.1	39	0.7	1.7
New Aurora/English Turn	5	10	6,230	10,076	33	37	23.5	4.6	28.1	23.3	0.4	0.5
New Orleans East	83	44	100,850	33,666	38	39	2.6	3.7	33.4	33	0.4	0.7
Uptown	96	86	36,750	27,533	38	39	46	34.7	38.1	38.5	1.3	1.6
Venetian Isles	2	0	1,822	0	24	0	43.6	0	50.1	0	0.5	0
Village De L’Est	3	1	7,201	483	39	34	1.8	0	38.5	50.9	0.2	1
No address provided	41	—	50,022	—	—	—	—	—	—	—	—	—
Total	788	613	487,211	235,652	—	—	—	—	—	—	—	—

Abbreviation: —, not applicable.

a Number of residents with annual income below the federal poverty level.

Hospitalizations at the block group-level for substance abuse disorders were higher in high-poverty block groups (block groups with a high percentage of residents at or below the federal poverty level) in both periods (*P* < .001) than in other block groups. Pre-Katrina, none of the neighborhood predictors were associated with these hospitalizations except for high poverty (*P *<.001). Post-Katrina, blighted block groups with nondisplaced residents (as measured by the number of households receiving mail in 2008) were significantly associated with higher rates of hospitalizations for substance abuse disorders. Block groups with a greater percentage of nonwhite residents and with water depths above the mean for that block group were associated with a high risk of substance abuse disorders, whereas areas with evidence of a greater percentage of removals of blighted properties (completed demolitions) had a lower risk of substance abuse disorders hospitalizations (Table 2).

**Table 2:** US Census Block Group Variables Associated With 1,401 Hospitalizations for Substance Abuse Disorders, New Orleans, 2004 and 2008

Variable	Estimate	Standard Error	*z* Value	Exponential	*P* Value
**2004**					
Intercept	−6.448	0.272	−23.680	0.002	<.001
Housing units, 2000^a^	−0.000	0.000	−1.472	1.000	.14
Population below FPL, %^b^	0.010	0.004	2.678	1.010	.007
White race, %	0.000	0.002	0.133	1.000	.89
Flood depth^c^	−0.053	0.029	−1.851	0.948	.06
Blighted buildings, %	0.016	0.016	0.989	1.016	.32
Building demolitions^d^, %	−0.008	0.014	−0.593	0.992	.55
**2008**					
Time 2008^e^	−0.419	0.313	−1.337	0.658	.18
Housing units, 2000^a^	0.001	0.000	2.402	1.001	.02
Population below FPL, %^b^	0.014	0.004	3.270	1.014	<.001
Population white race, %	−0.011	0.002	−5.072	0.989	<.001
Flood depth^c^	−0.128	0.031	−4.117	0.880	<.001
Imminent health threat	0.092	0.017	5.267	1.096	<.001
Building demolitions^d^, %	−0.052	0.023	−2.285	0.949	.02
Houses receiving mail^f^, %	0.003	0.001	2.544	1.0027	.01

Abbreviation: FPL, federal poverty level.

a Number of housing units obtained from the 2000 US Census.

b Percentage of people living below federal poverty level.

c Water depth as measured by the flood depth map obtained on August 29, 2005.

d Percentage of completed building demolitions from 2006 through 2009.

e The time variable, which differentiates the 2 years, indicating whether hospitalization rates were different in 2004 versus 2008.

f Households receiving mail by June 2008 per the US Postal Service.

### Clusters of substance abuse disorders

**Figure 1:**
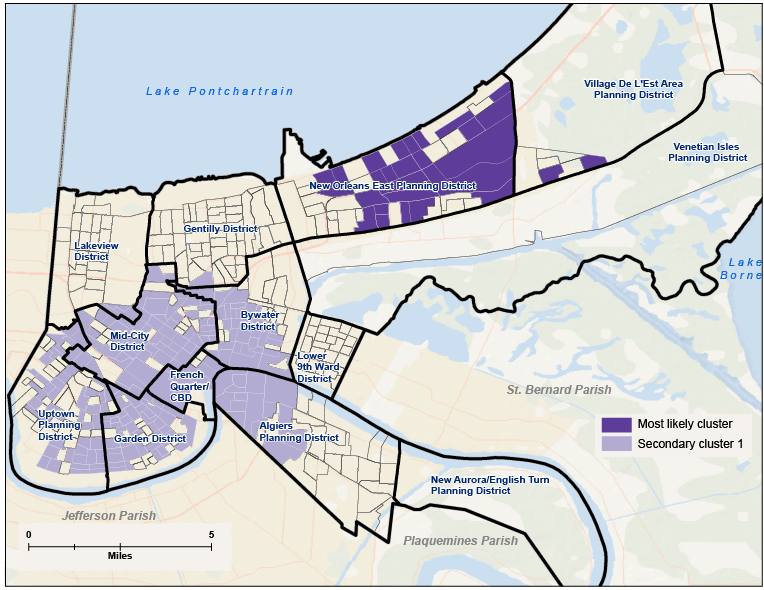
Pre-Katrina significant clusters of hospitalizations for substance abuse disorders at the block group level calculated by using the discrete Poisson model with sex and age group as covariates, New Orleans, 2004. The most likely cluster (the cluster least likely to be due to chance) was the New Orleans East area with 316 block groups and a relative risk of .38 compared with the city as a whole. The next highest was central New Orleans with a relative risk of 1.83.

After adjustment for age and sex of people hospitalized pre-Katrina for substance abuse disorders, the discrete Poisson model detected 2 significant clusters of substance abuse disorders (Table 3, Figure 1). The most likely cluster was in block groups that make up the New Orleans East District, consisting of 316 block groups with a relative risk of 0.36 compared with the rest of the city. That means that the New Orleans East area in 2004 had lower rates of substance abuse disorders than expected based on population and after controlling for sex and age. We found a secondary significant cluster that comprised multiple planning districts and was located in the block groups of Algiers, the Bywater, the Garden District, the French Quarter and Central Business District (CBD), and Uptown with a relative risk (RR) of 1.77 compared with the rest of New Orleans.

**Table 3:** Clusters of US Census Block Groups With Substance Abuse Disorders Measured by Using the Discrete Poisson Model, New Orleans, 2004 and 2008

Cluster	Observed Hospitalizations	Expected Hospitalizations	RR	LLR	*P* Value
**2004**					
Most likely cluster, (New Orleans East)	114	272.4	0.36	71.0	<.001
Secondary cluster 1, (Algiers, Bywater, Garden District, French Quarter, CBD)	655	477.0	1.77	51.1	<.001
**2008**					
Most likely cluster, (Eastern corner of Mid-City)	820.47	0.01	831.23	40.0	<.001
Secondary cluster 1, (Lower 9th Ward)	11.60	1.29	11.91	23.2	<.001
Secondary cluster 2, (Algiers)	0.44	96.86	0.40	22.1	<.001
Secondary cluster 3, (Southeast corner of Mid-City)	23.14	0.26	23.38	13.1	<.001

Abbreviations: RR, relative risk; LLR, log-likelihood ratio; CBD, Central Business District

**Figure 2:**
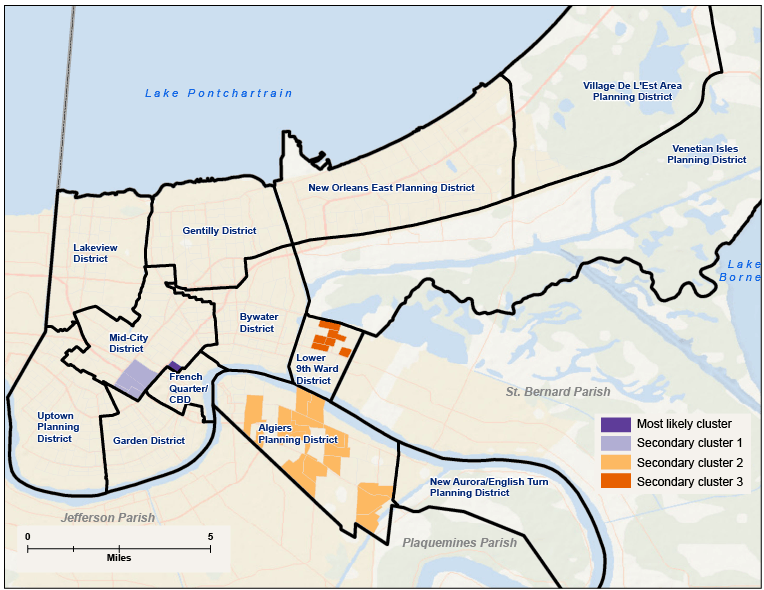
Clusters of hospitalizations for substance abuse disorders at the block group level calculated by using the discrete Poisson model with sex and age group as covariates, New Orleans, 2008. The most likely cluster was the eastern corner of Mid-City (relative risk [RR], 831.23). The next highest, in order, were the north corner of the Lower 9th Ward (RR = 11.91), Algiers (RR = 0.40), and the southeast corner of Mid-City (RR = 23.38).

Post-Katrina, there were 4 significant clusters of hospitalizations for substance abuse disorders in the city (Table 3, Figure 2). The most likely cluster was located in the eastern corner of Mid-City, the neighborhood bordering the French Quarter/CBD, including one Mid-City block group with a relative risk of 831.23 compared with the rest of New Orleans. Secondary clusters were located in the north corner of the Lower 9th Ward (RR = 11.91), Algiers (RR = 0.40), and the southeast corner of the Mid-City planning districts (RR = 23.38).

## Discussion

We described the geographic variation in hospitalizations for substance abuse disorders before and after Hurricane Katrina in New Orleans. We also identified neighborhood-level predictors associated with hospitalizations. We found that the rate of hospitalizations for substance abuse disorders increased in the aftermath of Hurricane Katrina. This result is not surprising given that a large segment of the local population experienced trauma, which had the potential to increase hospitalization rates at the same time that the city’s population was reduced. These 2 factors accounted for the high hospitalization rates in areas that lost population. This view is supported by a 2006 US Department of Homeland Security report, *The First Year after Hurricane Katrina*, which confirmed the shift in the New Orleans population (22). This displacement resulted in local population shifts and was a major contributing factor to this finding and underscores the effect of population shifts on statistical calculations after disasters.

Post-Katrina, men continued to have higher rates of hospitalizations for substance abuse disorders than women. This finding suggests that men in the study area were more likely to be substance abusers both before and after Katrina. Another possible explanation is that men were unequally exposed to chronic stressors associated with the disaster, such as reduced socioeconomic status and loss of employment and income, which contributed to hospitalizations. Similarly, one study found increases in substance use in women aged 15 to 39 years and in men aged 30 to 49 years in New Orleans and among young women evacuees living in Houston, Texas (23). Therefore, prevention and treatment interventions targeted to sex and age groups should be considered to maximize positive outcomes in high-risk areas after disasters.

We found that geographic patterns of hospitalizations shifted in post-Katrina New Orleans. The relatively low pre-Katrina rates of hospitalization in New Orleans East, a low-income area that had high flood levels, disappeared post-Katrina. The moderately high rates of substance abuse hospitalizations before Katrina in the French Quarter and CBD and in nearby areas remained higher than rates in the rest of the city; however, post-Katrina, smaller areas such as block groups in the Mid-City District had very high rates of substance abuse hospitalizations. The Lower 9th Ward emerged as an area with high hospitalization rates post-Katrina, although it did not stand out pre-Katrina, and the Algiers region had relatively low rates compared with New Orleans as a whole. Gruebner and colleagues (16) showed that psychosocial problems among people affected by Hurricane Sandy clustered predominantly in neighborhoods that were in close proximity to exposed areas (areas near the ocean) in New York City. This differs from the findings here and may be related to local decisions and opportunities for departure from storm-affected areas post-disaster. Unlike the Hurricane Sandy situation, many people in the areas affected by Hurricane Katrina were displaced or relocated; some moved and never returned to the city. One surprising result is that the hospitalization clusters detected in parts of Uptown, Mid-City, the French Quarter/CBD, and the Garden District — districts that were less affected by flooding during Hurricane Katrina — disappeared post-Katrina. The small number of hospitalizations for substance abuse disorders in these 4 block groups prevented evaluation of trends in detail, and substance abuse disorders possibly remained about the same before and after Katrina in these areas. The shift in the spatial concentration of substance abuse disorders changed the relative risk values used to identify spatial clusters. This emphasizes the need for multivariable analysis and more detailed data collection to better identify processes that are related to changes in substance abuse disorders, an important consideration for future research.

The neighborhood predictors of hospitalizations for substance abuse disorders in New Orleans are generally consistent with those reported in the literature indicating that blighted properties and poor socioeconomic status may increase disease risk (24,25,26). The positive effect of removal of blighted properties in a neighborhood may be a sign of wide neighborhood revitalization, whereas the presence of blight may impose stress on residents, which may in turn increase hospitalizations. Improvement in neighborhood conditions (eg, housing conditions) may significantly improve health outcomes.

Our study had limitations. One limitation is the largely retrospective study design and associated reliance on secondary data. The study assumed that if a household was receiving mail in 2008, its residents stayed through Katrina or returned to the city. However, we did not verify whether any households that received mail in 2008 were uninhabited during the that period, nor did we verify whether the hospital patients in the database of the Louisiana Department of Health and Hospital’s Bureau of Policy Research and Health Systems Analysis were those who stayed or returned versus those who were permanently displaced. Postal data can be unreliable because people not living in a neighborhood may collect mail. This is especially true for 2008, because the city at that point had not fully recovered. For example, during this time, neighborhoods like the Lower 9th Ward were at less than 10% occupancy (23). Also, our study relied exclusively on hospital admissions data that could potentially lead to selection bias or hospital access bias. A bias plausible in the current study is due to the variation in hospitalizations post-Katrina, because it was likely that a disproportionate number of residents were hospitalized in New Orleans hospitals relative to hospitals in neighboring towns and cities. We also calculated hospitalization rates based on Encounter ID (https://www.hl7.org/fhir/encounter.html), which allowed separate admissions by the same patient to be linked. Although this allowed us to identify individual patients, we used only the initial visit in our analysis because it was not possible to identify an eligible hospital readmission from the convoluted administrative claims data, and data often did not include a discharge date.

This study demonstrates that spatial analysis techniques, GIS, and statistical methods can be used to identify at-risk populations, delineate at-risk areas, and identify residential context risk factors before and after a disaster. This information can help those working in disaster response to design, plan, and deploy post-disaster interventions in a spatially targeted way. The approach used in the current study is generalizable to other disasters and to other types of psychological vulnerabilities.

## Appendix ICD-Codes Used in Spatial Analysis of Hospitalizations for Substance Abuse Disorders, New Orleans, 2004 and 2008

**Table:**

SLCCS Code	SLCCS Description^a^	ICD-9 Code
66	Alcohol-related mental disorders	2910, 2911, 2912, 2913, 2914, 2915, 2918, 29181, 29189, 2919, 30300, 30301, 30302, 30303, 30390, 30391, 30392, 30393, 30500, 30501
67	Substance-related mental disorders	2920, 29211, 29212, 2922, 29281, 29282, 29283, 29284, 29289, 2929, 30400, 30401, 30402, 30403, 30410, 30411, 30412, 30413, 30420, 30421, 30422, 30423, 30430, 30431, 30432, 30433, 30440, 30441, 30442, 30443, 30450, 30451, 30452, 30453, 30460, 30461, 30462, 30463, 30470, 30471, 30472, 30473, 30480, 30481, 30482, 30483, 30490, 30491, 30492, 30493, 3051, 30520, 30521, 30522, 30523, 30530, 30531, 30532, 30533, 30540, 30541, 30542, 30543, 30550, 30551, 30552, 30553, 30560, 30561, 30562, 30563, 30570, 30571, 30572, 30573, 30580, 30581, 30582, 30583, 30590, 30591, 30592, 30593, V1582

Abbreviation: ICD-9, *International Classification of Diseases, 9th Revision*; SLCCS, single layer clinical classification system.

^a^ Disease definitions are based on the SLCCS, which is used to group the different *ICD-9-Clinical Modification* codes to map a particular disease (https://www.hcup-us.ahrq.gov).
